# Degradation of polyethylene plastic bags and bottles using microorganisms isolated from soils of Morogoro, Tanzania

**DOI:** 10.3389/fmicb.2022.1077588

**Published:** 2022-12-19

**Authors:** Monica D. Nakei, Gerald Misinzo, Hamisi Tindwa, Ernest Semu

**Affiliations:** ^1^Department of Soil Science, Sokoine University of Agriculture, Morogoro, Tanzania; ^2^Department of Veterinary Microbiology, Parasitology and Biotechnology, College of Veterinary Medicine and Biomedical Sciences, Sokoine University of Agriculture, Morogoro, Tanzania

**Keywords:** actinomycetes, bacteria, fungi, biodegradation of plastics, polyethylene

## Abstract

Plastics are of great significance in today’s world due to their extensive use such as packaging food and carrying other goods, which have improved the quality of human life. However, plastics have low biodegradability and are persistent in the environment, becoming a major source of pollution. With regard to the current methods used in the management of plastic wastes, the degradation of plastics using beneficial soil microorganisms has recently gained attention due to their ability to degrade different types of plastics including polyethylene (PE) polymers. The study herein was conducted to isolate and identify microorganisms from agricultural soils capable of degrading plastics. Soil samples were inoculated into nutrient, potato dextrose, and starch-casein agar for the isolation of bacteria, fungi, and actinomycetes, respectively. During isolation, fungi and bacterial plates were incubated for 5 days and for 14 days, respectively. The population of bacteria ranged from 1 × 10^5^ to 1.21^5^ × 10^5^ and that of fungi from 1.60^4^ × 10^4^ to 8.6 × 10^4^ whereby actinomycetes ranged from 1.04^5^ × 10^5^ to 2.99^5^ × 10^5^ CFU/g of soil. However, the tested microorganisms showed significant (*p* ≤ 0.05) differences in the ability to degrade PE bags and bottles as depicted by the diameters of clear zones around the colonies. The diameters of clear zones ranged from 19.3 to 47.5 mm and 25.9 to 32.2 mm after 17 days for bacteria and actinomycetes, respectively, and those of fungi ranged from 30.0 to 66.3 mm after 13 days. Among the bacteria, actinomycetes, and fungi, unsequenced bacterial and actinomycete isolates B1 and A3 as well as *Aspergillus* sp. (F7) were the most efficient degraders of PE plastic bags. This retrospective study sheds light on our understanding and the need for the bioprospecting of agricultural soils, water bodies, and landfills containing plastic wastes that could lead to the identification of more efficient microbial species with the ability to degrade plastics.

## 1 Introduction

Plastics are of great significance in today’s world due to their widespread use, which has enabled improvement in the quality of human life through the ease of packaging of foods and other items, thus lengthening their shelf life (Ilyas et al., 2018). Due to its demand, the global yield of plastics reached 368 million tons per annum in 2019, and this figure is expected to double over the next 20 years (Geyer et al., 2017; Plastic Europe, 2018). China and the European Union account for 29.4 and 18.5% of all the plastics used in the world, respectively (Plastic Europe, 2018; Ru et al., 2020). The mainly manufactured and widely used plastic types are polyethylene (PE) (36%), polypropylene (PP) (21%), and polyvinyl chloride (PVC) (12%), polyethylene-terephthalate (PET) 10%, polyurethane (PU) 10%, and polystyrene (PS) 10%, and others (Geyer et al., 2017). However, all these plastics are high molecular weight polymers whose biodegradability is low (Burke et al., 2012). Hence, plastics are persistent once introduced, after use, into the environment and are one of the sources of environmental pollution (Ostle et al., 2019). Their single-use and disposal both on land and in aquatic environments have resulted in their accumulation due to less, if any, biodegradation, making the environment unaesthetic, with possible health implications to humans, animals, and other organisms (Zalasiewicz et al., 2016; Bergmann et al., 2019; Jamieson et al., 2019; Ali et al., 2021a). Moreover, plastic polymers and additives that are frequently blended into commercial-grade plastics have also been shown to accumulate in marine species harvested for human consumption (Markic et al., 2018; Webb et al., 2019). In addition, it has been projected that up to 26 billion tons of plastic waste are likely to be produced by 2050 (Ru et al., 2020) and more than half to be thrown away into landfills that finally enter ecosystems such as oceans and lakes which will, perhaps, worsen the situation.

Currently, several methods including landfilling, incineration, and mechanical and chemical recycling have been used to dispose plastic waste (Ru et al., 2020). It is reported that between 9 and 12% of global waste is recycled and incinerated while 79% is discarded into landfills or the natural environment (Geyer et al., 2017). Landfilling is the preferred method of disposing plastic wastes in developing countries due to its low cost. However, the accumulated plastic wastes in landfills continue to occupy vast land that could be put to other uses. Incineration of plastic wastes can reduce the demand for landfills and recover heat energy, but this process generates secondary pollutants such as dioxins, carbon monoxide, and nitrogen oxides into the environment (Lear et al., 2021). Although mechanical recycling has been applied for reusing thermoplastic wastes, the properties of most recycled materials are compromised and the commercial value of resulting products is limited (Ru et al., 2020). As an alternative, chemical recycling has the potential to recover monomers and other chemicals from plastic wastes, but its success relies on the affordability of processes and the efficiency of catalysts (Rahimi and Garciá, 2017). These studies demonstrate that there is a need to explore an innovative recycling method to dispose plastic wastes.

Microorganisms from different ecosystems including agricultural soils, aquatic environments, and landfills have been observed to degrade different types of synthetic plastics (Ali et al., 2021b). Some of the identified microorganisms include bacteria such as *Bacillus, Pseudomonas*, *Azotobacter*, *Ralstonia*, and *Halomonas* spp. (Biki et al., 2021). *Pseudomonas* spp. can degrade low-density polythene (LDP), PP, PE, and nylon (Nanda et al., 2010; Pramila, 2012; Gupta and Devi, 2020; Skariyachan et al., 2021). Various species of *Streptomyces* have also been associated with the degradation of low-density polyethylene (LDPE) with varying degrees (Gupta and Devi, 2020). Examples of fungal degraders of PE, LDPE, and high-density polyethylene (HDPE) include *Aureobasidium*, *Rhodotorula, Kluyveromyces*, and *Aspergillus* (Muhonja et al., 2018; Aderiye et al., 2019). These studies provide evidence that several microorganisms are capable of degrading synthetic plastics. Although several studies on the biodegradation of plastic have been carried out, many have focused on the biodegradation of a single kind of plastic and there is a need to identify those with the potential to degrade different kinds of plastics.

The degradation of synthetic plastics by soil microorganisms is gaining the attention of different researchers (Ali et al., 2021a). Beneficial and non-pathogenic soil microorganisms that degrade organic matter in the soil are potential degraders of different types of synthetic plastic wastes, which enter agricultural soils. To achieve this it needs a careful isolation and identification of effective species from different groups of fungi, bacteria, and actinomycetes from different soil types including those with low carbon content. The present study focused on the determination of the potential of bacteria, actinomycetes, and fungi isolated from the soils of Morogoro, Tanzania, to degrade different polyethylene plastic bags and bottles and characterize the effective species based on the morphological and genetic attributes. The findings of this study will contribute to enriching the knowledge which the researchers could tap into, for the benefit of further studies, on the management of plastic waste through degradation by beneficial and non-pathogenic microorganisms recovered from agricultural soils. Since these microorganisms are not potential plant pathogens, the identified fungi, bacteria, and actinomycetes in this study can be used in agricultural soils to get rid of plastics, which hinder plant root growth and water movement.

## 2 Materials and methods

### 2.1 Survey for identification of dominant plastic wastes, collection of soil samples, and polyethylene plastic bags and bottles

Dominant plastic wastes in the study site were identified after surveying the area. During the survey, the plastics on the soil surface and sub-surface were observed to undergo degradation with time as evidenced by the strength of plastic material when broken or smashed by hands. The dominant plastic wastes were PE plastic bags and bottles. Plastic bags are used as temporary packaging materials after purchasing goods or food while plastic bottles are mainly used for packaging drinking water from industries. For uniformity in the degradation of plastics by microorganisms, plastic bags, and bottles of the same type (dominant plastic wastes) were purchased to conduct this experiment. Soil samples for isolating plastics-degrading microorganisms were collected in the vicinity of the Morogoro-Iringa Road at Kasanga village and within Sokoine University of Agriculture (SUA) maize fields, close to the Department of Agricultural Economics and Agribusiness building. The university is located at latitude 06°50′ S and longitude 37°38′ E and an altitude of 526 m above sea level (m.a.s.l). In total, four locations, three at Kasanga village (Kasanga 1, 2, and 3) and one at SUA farm were identified for soil sampling within the study site. These included sites that harbored plastic waste for different lengths of time, as well as those that visibly had not encountered any plastic waste. Kasanga 2 and 3 harbored plastics for about 10 years while SUA fields for 5 years but the deposition time depends on the time of plastic disposal. Kasanga 1 had few plastic pollutants most likely blown by the wind since the area is not exhibited with human activities. Kasanga 2 had less visible plastic wastes since the site is somehow far from the shops while Kasanga 3 had many plastic wastes since the place has many shops, a fresh vegetable market, a small market, and small local food courts as well as smallholder brick making enterprises. The SUA farm (maize fields) had many visible plastic wastes blown by the wind from the Mafiga village waste damping area in use for about 15 years. The soils with physical contact with plastics were sampled at 5–10 cm depth. Surface soils exposed to sunlight were not collected because the degradation of plastic wastes might be due to ultraviolet (UV) radiation instead of microorganisms (Volke-Sepulveda et al., 2002). The soil samples were transported to the Soil and Geological Sciences laboratory at SUA for the isolation of actinomycetes, bacteria, and fungi. Immediately upon the sample’s arrival in the laboratory, soil samples were stored at 4°C in the refrigerator before isolation.

### 2.2 Isolation of bacteria, fungi, and actinomycetes from the soil samples

A total of 1 g of soil sample was suspended in 99 ml of sterile distilled water and afterward incubated at 28°C (Usha et al., 2011) on a rotary shaker at 150 runs per minute (rpm) (Siddique et al., 2014) for 30 min. Then, 10-fold dilutions from 10^–1^ to 10^–6^ of soil samples were prepared as previously described by Usha et al. (2011). Nutrient agar (NA) was prepared for the isolation of bacteria as described by Akmar et al. (2011). Potato dextrose agar (PDA) was used to isolate fungi from the soil following the procedure described by Kaiding and Kumar (2018). Starch-casein agar (SCA) was prepared for the isolation of *Streptomyces* as previously described by Balakrishna et al. (2012). Approximately, 1 ml aliquots of the 10^–3^–10^–6^ suspensions were dispensed into sterile Petri dishes in triplicates followed by the addition of 15 ml of the molten media. The Petri dishes were then swirled gently to uniformly mix the inoculum with media and left to solidify at room temperature (25 ± 2°C). The plates for isolation of bacteria and fungi were incubated upside down at 30°C for 5 days for colonies to develop while those for the isolation of actinomycetes were incubated upside down at 30°C for 14 days. The plate count method was used for enumeration of microbial colonies per gram of soil sample. The obtained colonies were subcultured repeatedly in the respective media to obtain pure cultures of the microbes, which were subsequently preserved at 4°C.

### 2.3 Morphological characterization of the microorganisms

Actinomycetes and bacterial isolates were morphologically characterized based on the color and edges of their colonies, cell shapes, and Gram-stain reactions (Tachibana et al., 2010). The fungal isolates were characterized based on the back and front colony color and sporulation. The fungal isolates were stained using lactophenol cotton blue solution as described by Mathew et al. (2016) and Maitig et al. (2018). A drop of lactophenol cotton blue solution was placed on a slide, using an inoculating needle/loop, followed by careful spreading of a fungal culture to obtain a thin preparation on the slide. A coverslip was afterward placed on the drop and gently lowered to avoid air bubbles and left for about 5 min. The slides were observed under a light microscope (Olympus CX43) at 1,000× magnification with low power for screening in low intensity as previously described by Golding et al. (2016). The images of the colonies were captured using a digital camera (Nikon, Hong Kong, China) mounted onto the light microscope.

### 2.4 Molecular identification of selected microorganisms

The DNA was extracted from pure colonies of actinomycetes, bacteria, and fungi using mini-spin columns (Qiagen, Hilden, Germany), as per the manufacturer’s instructions. Bacteria, actinomycetes, or fungi were digested using proteinase K (20 mg/L) for 3 h at 56°C. Digestion was followed by lysis and precipitation of proteins by heating at 56°C for 15 min and the addition of ethanol, respectively. The DNA was passed through the positively charged silica columns, washed using buffers, and eluted using nuclease-free water. The DNA was stored at –20°C until further use for polymerase chain reaction (PCR). Amplification of 16S rDNA of actinomycetes and bacteria was performed using universal 27F and 1492R primers, as previously described by Isik et al. (2014). The PCR amplification conditions included an initial denaturation at 95°C for 10 min, followed by 40 cycles of denaturation at 95°C for 1 min, annealing at 62°C for 30 s, and extension at 72°C for 30 s, followed by a single final extension at 72°C for 10 min. The amplification of 5.8S rDNA and flanking ITS regions of fungi was performed using ITS1 and ITS4 primers (Iwen et al., 2002). The PCR amplification conditions included an initial denaturation at 95°C for 10 min, followed by 40 cycles consisting of denaturation at 95°C for 30 s, annealing at 57°C for 45 s and extension at 72°C for 1 min, and a final extension at 72°C for 10 min. The amplified DNA fragments were separated by electrophoresis using a 1.2% agarose, visualized, and imaged using a gel documentation system after staining with GelRed. PCR amplicons resulting from 5.8S rDNA and 16S rDNA amplification were sequenced using the dideoxynucleotide cycle sequencing method on an ABI 3500 genetic analyzer (Applied Biosystems, Foster City, CA, United States).

### 2.5 Degradation of polyethylene plastic bags and bottles of plastics by actinomycetes, bacterial isolates, and fungal isolates

The ability of the actinomycetes, bacterial isolates, and fungal isolates to degrade different types of plastics was carried out using the Bushnell and Haas mineral agar medium (Bushnell and Haas, 1940) supplemented with different types of plastics. The PE powder was obtained from ground plastics sieved through a 0.6-mm sieve. After sieving, 1 g of PE powder was added to 1,000 ml (0.1% w/v) of this mineral salt medium and mixed for 1 h at 120 rpm using a shaker. The pH of the medium was adjusted to 7.0 ± 0.2 and autoclaved at 1.05 Kg/cm^2^ at 121°C for 15 min. The medium was left to cool to 50°C and dispensed into Petri dishes until the solidification of the media. The isolated microorganisms were transferred onto the plates and incubated at 27°C for up to 21 days while periodically observing for the formation of clear zones around the colonies, which evidenced the plastic degradation. The diameters of such colonies and the clear zones formed around them were measured using a ruler. Cultures that had larger clear zones were selected and tested for their comparative efficiency in degrading the plastics using the completely randomized design (CRD) with four replications in the Bushnell and Haas medium. The diameters of clear zones around the colonies were measured on days 5, 7, 9, 11, and 13 for fungi and on days 5, 8, 11, 14, and 17 for bacteria and actinomycetes.

## 3 Statistical analysis

The diameter of clear zones for each isolate of actinomycetes, bacteria, and fungi was subjected to the analysis of variance to test the significance of variation due to site, isolates, time, and their interactions. Analysis was performed using GenStat (15th Edition). Differences among treatment means were separated using Tukey’s *post-hoc* tests at *p* ≤ 0.05. Actinomycetes and bacteria were identified based on 16S rDNA nucleotide sequencing followed by nucleotide identity search at GenBank using the Basic Local Alignment Search Tool (BLAST). Nucleotide sequencing and identity search using BLAST of 5.8S rDNA and the flanking intergenic spacer regions (ITS1 and 2) were used to identify the fungi. The quality of nucleotide sequences was analyzed visually using the sequence scanner v.1.0 (Applied Biosystems, Foster City, CA, United States). The nucleotide sequences obtained using forward primers were overlapped with reverse complement sequences of reverse primers using notepad (Microsoft Windows 8.1, 2013). The nucleotide sequences obtained were used to search for the similarity to other publicly available nucleotide sequences at GenBank using BLAST. The identities of the actinomycetes, bacteria, and fungi were inferred based on the highest nucleotide identity following BLAST.

## 4 Results

### 4.1 Isolated microorganisms

Table 1 shows the total counts of microbial populations of the soils used in the present study. The population of actinomycetes was higher than that of bacteria and fungi whereby the fungi population was lower than that of bacteria.

**TABLE 1:** Microbial populations of the studied soils.

Soil	CFU/g soil		
	Bacteria	Fungi	Actinomycetes
Kasanga 1 (few plastics)	1.00 × 10^5^	3.73 × 10^4^	1.04 × 10^5^
Kasanga 2 (few plastics)	1.12 × 10^5^	8.10 × 10^4^	1.34 × 10^5^
Kasanga 3 (many plastics)	1.00 × 10^5^	1.60 × 10^4^	2.99 × 10^5^
SUA farm (many plastics)	1.21 × 10^5^	4.70 × 10^4^	1.57 × 10^5^

### 4.2 The abilities of isolated actinomycetes, bacteria, and fungi in degrading polyethylene plastic bags

The abilities of the microorganisms from the soils of Kasanga 1, Kasanga 2, Kasanga 3, and SUA farm to degrade plastic bags are shown in Table 2. Clearly, different isolates showed differences in abilities to degrade ground plastic bags as depicted by the differences in the diameters of clear zones surrounding the colonies. Bacterial isolates from the soil collected from the SUA farm recorded the smallest clear zone diameter of 1 mm and the largest diameter of 54 mm. The smallest average clear zone diameter was recorded in isolates from Kasanga 1 soil, and the largest one, 27.8 mm, was in isolates collected from the SUA farm soil. Fungal isolates from Kasanga 3 displayed a minimum clear zone diameter of 5.3 mm while a maximum diameter of 66.0 mm was recorded in an isolate from Kasanga 1 soil. The smallest average clear zone diameter, 27.7 mm, was recorded in fungal isolates from Kasanga 2 soil while the largest one, 39.1 mm, was in fungal isolates from Kasanga 1 soil. On the other hand, the isolates of actinomycetes collected from Kasanga 2 soil displayed the smallest clear zone diameter, 11.0 mm; whereas, the largest one, 58.3 mm, was recorded in isolates from Kasanga 2 soil. Actinomycete isolates from the SUA farm soil showed the smallest average clear zone diameter, 25.9 mm, while those from Kasanga 1 soil displayed the largest clear zone diameter, 35.3 mm.

**TABLE 2:** Screening the abilities of isolated bacteria, fungi, and actinomycetes from four different soils in degrading ground plastic bags.

Soil + organism	Number of isolates tested	Number showing biodegradation ability	Diameter of clear zone			SD
			Smallest (mm)	Largest (mm)	Mean	
**Kasanga 1**						
Bacteria	14	13	9.7	32.0	15.1	5.6
Fungi	13	13	28.7	66.0	39.1	10.2
Actinomycetes	5	5	22.0	52.0	35.3	12.0
**Kasanga 2**						
Bacteria	–	–	–	–	–	–
Fungi	5	5	15.7	57.7	32.6	24.1
Actinomycetes	20	19	11.0	58.3	27.2	18.2
**Kasanga 3**						
Bacteria	16	16	9.7	34.0	16.6	5.9
Fungi	16	14	5.3	52.7	27.7	15.8
Actinomycetes	5	5	22.0	51.0	34.8	12.6
**SUA farm**						
Bacteria	11	11	1.0	54.0	27.8	16.2
Fungi	8	8	22.0	57.0	33.2	19.1
Actinomycetes	7	7	36.0	52.9	25.9	24.7

(–), not observed; SD, standard deviation.

### 4.3 The abilities of isolated actinomycetes, bacteria, and fungi in degrading polyethylene plastic bottles

The abilities of the microorganisms to degrade ground plastic bottles are presented in Table 3. Among microbial groups of actinomycetes bacteria and fungi, there were clear differences in their abilities to degrade the ground plastic bottles. Bacterial isolates from the soil collected Kasanga 1 recorded a minimum clear zone diameter of 1 mm whereas a maximum diameter of 56 mm was observed in a bacterial isolated from the SUA farm soil. Moreover, the smallest average clear zone diameter, 12.8 mm, was observed in bacterial isolates from Kasanga 1 soil while the highest zone, 30.0 mm, was displayed by bacterial isolates from the SUA farm soil. Fungal isolates from Kasanga 3 displayed a minimum clear zone diameter of 5 mm while a maximum diameter of 73.7 mm was achieved in an isolate from Kasanga 2 soil. Fungal isolates from Kasanga 3 soil displayed the smallest average diameter of clear zone, 23.3 mm with the largest one, 48.2 mm being in isolates obtained from SUA farm soil. On the other hand, the minimum diameter of the clear zone, 10.0 mm, was observed in the actinomycete isolate from Kasanga 1 while the maximum of 60.0 mm in the isolate was collected from Kasanga 2 soil. Actinomycetes from the SUA farm soil displayed the smallest average diameter of the clear zone (30.0 mm) while the largest one (36.9 mm) was in isolates collected from Kasanga 2 soil.

**TABLE 3:** Screening the abilities of isolated bacteria, fungi, and actinomycetes from four different soils in degrading ground plastic bottles.

Soil + organism	Number of isolates tested	Number showing biodegradation ability	Diameter of clear zone			SD
			Smallest (mm)	Largest (mm)	Mean	
**Kasanga 1**						
Bacteria	14	13	1.0	42.7	12.8	10.5
Fungi	13	13	16.7	55.0	39.7	12.4
Actinomycetes	5	5	10.0	48.0	32.2	10.8
**Kasanga 2**						
Bacteria	–	–	–	–	–	–
Fungi	5	5	47.3	73.7	59.3	11.1
Actinomycetes	20	19	30.0	60.0	36.9	14.8
**Kasanga 3**						
Bacteria	16	16	5.0	46.0	17.3	8.9
Fungi	16	14	5.0	58.7	23.3	18.3
Actinomycetes	5	5	22.0	43.0	32.3	8.8
**SUA farm**						
Bacteria	11	11	8.7	56.0	30.3	16.7
Fungi	8	8	12.0	66.7	48.2	19.5
Actinomycetes	7	7	16.0	43.0	30.0	10.1

(–), not observed; SD, standard deviation.

### 4.4 Morphological characterization of plastic-degrading microorganisms

**FIGURE 1:**
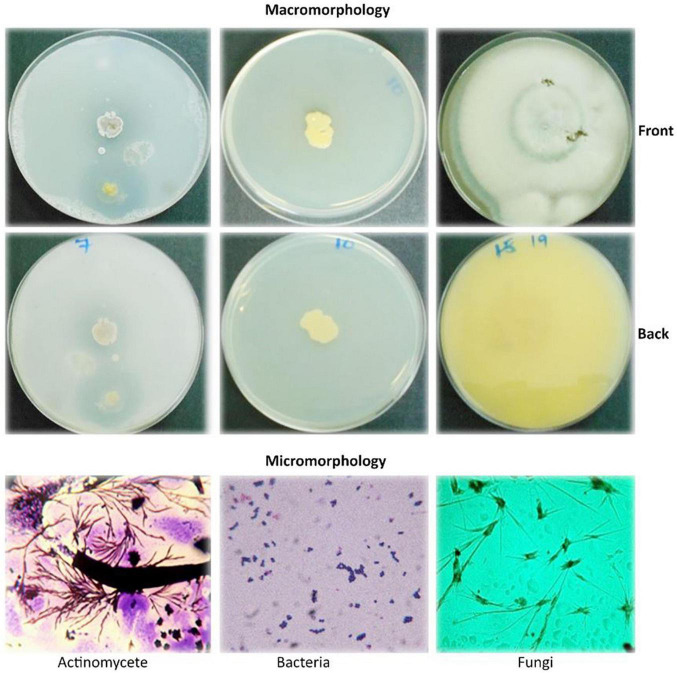
Macromorphological and micromorphological characteristics of the actinomycetes, bacteria, and fungi studied for the degradation of plastics.

The macro- and micromorphological features of the actinomycete, bacterial, and fungal isolates as examined on the culture plates with the naked eye and under the light microscope, respectively, are presented in Figure 1. Generally, the actinomycetes were mainly large dry colonies, with the colors of aerial mycelia varying from white to grayish to blue-gray, with the reverse color almost brownish for all actinomycetes. Bacterial colonies were slimy and shiny on the surface, with whitish to yellow colors. Fungal colonies were more profuse, with substantial sporulation. The microscopic features showed actinomycetes and fungi to be filamentous, but bacteria to be single-celled entities.

The macroscopic and microscopic features of bacteria and actinomycetes are summarized in Table 4. The shapes of the colonies, cells, color, and the Gram reaction for most of the bacterial isolates varied from one to another. The color of the colonies varied from yellow to white, with small to relatively large colonies. The cells varied from cocci to rod chains and/or groups. The rod-like bacteria could be of the genus *Bacillus*. The colony characteristics of the actinomycetes varied in appearance/color but were compact in size. The colors of the colonies varied from white, grayish, to bluish, and the mycelia were the characteristic of the genus *Streptomyces* when grown/cultured in SCA appeared dry on the surface.

**TABLE 4:** Morphology of plastic-degrading bacteria and actinomycetes.

Isolate	Colony morphology	Shape	Color	Gram stain
**Bacteria**				
B1	White, large, irregular shape	Large rods with ovoid ends	Purple	+
B2	Yellow, large with raged ends	Cocci in short chains	Purple	+
B3	White, small, round	Cocci rods	Pink	–
B4	Yellow, small	Cocci in groups	Pink	–
B5	White, small	Cocci in groups	Purple	+
B6	White, large, raged ends	Cocci in tetrads	Purple	+
B7	White, large, irregular shape	Large rods with ovoid ends	Purple	+
B8	White, small	Cocci in groups	Purple	+
**Actinomycetes**				
A1	Whitish, large, and dry colony	Filamentous	Gram+	
A2	White, large, and dry	Filamentous	Purple	+
A3	White to dark, large, and dry	Filamentous	Purple	+
A4	Grayish, large, and dry	Filamentous	Purple	+
A5	Whitish with a brown shadow, large, and dry ring	Filamentous	Purple	+
A6	Bluish, large, and dry	Filamentous	Purple	+
A7	Whitish, large, and dry	Filamentous	Purple	+
A8	Whitish, large, and dry	Filamentous	Purple	+

Under the microscopic observations, some of the isolates developed a black substrate mycelium with sporulated aerial mycelium. The detailed morphological features of fungi are summarized in Table 5. The colors of the colonies of the fungi varied from white-brown, grayish-brown, to deep green in the front side of the colonies, with moderate sporulation, while the reverse of the colonies for most of the cultures showed a deep dirty brown color.

**TABLE 5:** Morphology of plastics-degrading fungi.

Isolate code	Colony morphology		Sporulation	Microscopic features
	Front	Back		
F1	Grayish-green, cotton like	Brownish color	Moderate	Smooth walls conidia which are globose to subglobose with phialides like ampulliform
F2	Grayish-brown, powdery/granular	Brownish color	Moderate	Conidia a produced in abundance within the pycnidia on narrow thread-like phialides, which are pycnidial wall cell, Conidia globose to cylindrical
F3	Whitish-bluish, cotton like	Deep brownish	Moderate	Short conidiophores branching from one foot cell, globose to hemispherical vesicle, branched straight phialides
F4	White to brown	Deep dirty brown	Moderate	Long conidiophores branching from one foot cell, globose to hemispherical vesicle, branched straight phialides
F5	White to brown	Deep dirty brown	Moderate	Long conidiophores branching from one foot cell, globose to hemispherical vesicle, branched straight phialides
F6	Deep green with white periphery	Brown	Moderate	Conidia globose to subglobose, conidiophore are on the surface hyphae
F7	White to brown	Deep dirty brown	Moderate	Long conidiophores branching from one foot cell, globose to hemispherical vesicle, branched straight phialides
F8	White to brown	Deep dirty brown	Moderate	Long conidiophores branching from one foot cell, globose to hemispherical vesicle, branched straight phialides

### 4.5 Molecular identification of plastic-degrading microorganisms

**FIGURE 2:**
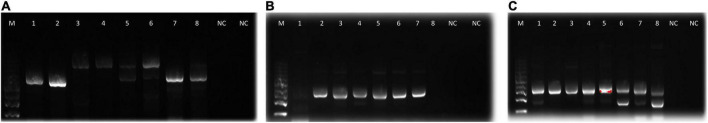
Agarose gel electrophoresis bands of PCR products from the microbial isolates **(A)**: fungi, **(B)**: actinomycetes, and **(C)**: bacteria. M, molecular weight marker; 1–8, bands for DNA samples; NC, negative control.

Amplification of 16S rDNA for bacteria and actinomycetes and of 5.8S rDNA and flaking ITS1 and ITS2 for fungi produced PCR products with sizes ranging between 300 and 800 bp (Figure 2). The DNA was of good quality and quantity for DNA sequencing. The identity of the species of bacteria, actinomycetes, and fungi based on 16S rDNA nucleotide sequences is shown in Table 6. The isolates that degraded plastics and their amplified DNA could not be sequenced are shown in Table 7. The sequenced isolates of bacteria, actinomycetes and fungi belonged to different species including *Bacillus cereus, Sinomonas* sp., and *Cellulosimicrobium* sp. while actinomycetes included *Streptomyces werraensis* and *S. rochei* (Table 6). The fungal isolates that degraded, on the other hand, belon*g*ed to different species including *Eupenicillium rubidurum*, *Phoma* sp., *Neosartorya fischeri*, *Aspergillus terreus*, *Aspergillus* sp., and *Talaromyces islandicus*. *Aspergillus terreus* appeared in two different soils of Kasanga 2 and SUA farm.

**TABLE 6:** Identity of plastic-degrading bacteria, actinomycetes, and fungi.

Current isolate/Strain	Location	Identification	Similarity of current isolate to GenBank		Type of plastic degraded
			Accession numbers	% identity	
**Bacteria**					
B2	SUA farm	*B. cereus*	KC683896	100	PE plastic bag
B3	SUA farm	*Sinomonas* sp.	HE793513	100	PE plastic bottle
B4	SUA farm	*Sinomonas* sp.	KJ504159	100	PE plastic bottle
B5	Kasanga 3	*Cellulosimicrobium* sp.	EU307933	100	PE plastic bottle
B6	Kasanga 3	*Cellulosimicrobium* sp.	LN846832	99	PE plastic bottle
**Actinomycetes**					
A2	Kasanga 3	*S. werraensis*	KM215730	99	PE plastic bag
A8	Kasanga 1	*S. rochei*	KF444515	100	PE plastic bottle
**Fungi**					
F1	Kasanga 3	*Eupenicillium rubidurum*	HQ608058	100	PE plastic bag
F2	Kasanga 1	*Phoma* sp.	EF423518	100	PE plastic bag
F3	Kasanga 3	*Neosartorya fischeri*	AF455538	99	PE plastic bag
F4	Kasanga 2	*A. terreus*	KC119206	100	PE plastic bottle
F5	Kasanga 2	*A. terreus*	KC119206	100	PE plastic bag
F6	SUA farm	*Talaromyces islandicus*	NR_103664	100	PE plastic bottle
F7	Kasanga 1	*Aspergillus* sp.	KF367546	100	PE plastic bottle
F8	SUA farm	*A. terreus*	KM491895	99	PE plastic bottle

**TABLE 7:** Microbial isolates that their DNA could not be sequenced.

Bacterial isolate	Location	Actinomycete isolate	Location
B1	Kasanga 1	A1	SUA farm
B7	Kasanga 2	A3	Kasanga 1
B8	Kasanga 2	A4	SUA farm
		A5	Kasanga 2
		A6	Kasanga 2
		A7	Kasanga 3

### 4.6 Comparative degradation of ground plastic bags by fungi (5–13 days), bacteria, and actinomycetes (5–17 days) isolates

**FIGURE 3:**
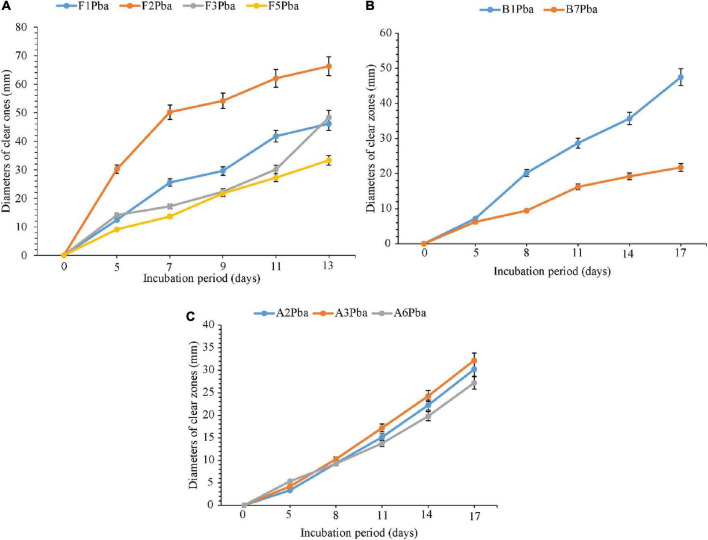
Comparative ability of the **(A)** fungi, **(B)** bacteria, and **(C)** actinomycetes in degrading ground plastic bags. Error bars represent the mean ± SD of three independent biological replicates (the names of species coded with letters F represent fungi, B represents bacteria, and A represents actinomycetes, and the numbers represent their positions in a particular group as shown in Table 6).

The comparative degradation of plastic bags by actinomycetes, bacteria, and fungi is shown in Figure 3. There was a clear variation in the diameters of clear zones for actinomycetes, bacteria, and fungi in degrading ground plastic bags on solidified Bushnell and Haas agar medium. There were differences in growth among species of fungi during the degradation of plastic bags from the 5th to the 13th day. The minimum diameter of 9.1 mm was observed in *Aspergillus terreus* (F5) during the 5th day and the maximum diameter of 30.2 mm was observed in *Phoma* sp. (F2). The growth continued to vary from the 7th, 9th, and 11th days and, finally, on the 13th day, the minimum clear zone diameter was 33.3 mm, which was observed in the same isolate which is *Aspergillus terreus* F5 and the maximum diameter was 66.3 mm, observed in the same species which was *Phoma* sp. F2 isolate. The clear zone diameter of other fungal isolates was intermediate. *Phoma* sp. F2 was the most efficient, and *Aspergillus terreus* F5 was the least efficient in degrading the ground plastic bags. The minimum clear zone diameter of 6.2 mm recorded by bacteria on the 5th day was observed in unsequenced isolate B7 and the maximum, 7.2 mm in B1. The variation in growth continued from the 8th, 11th, and 14th day; finally, on the 17th day, the minimum clear zone diameter of 21.7 mm was recorded in the same isolate B7 and the maximum, 47.5 mm in isolate B1. This indicates that the bacterial isolate B1 was efficient as the B7 in degrading ground plastic bags. In the case of actinomycetes, there was somehow unpredictable variation in the growth of colonies and the formation of clear zones. The variation started from the 5th day as in fungi and bacteria whereby the minimum clear zone diameter of 3.3 mm was recorded in *Streptomyces weraensis* (A2) while the maximum, 5.3 mm, was displayed by another unsequenced isolate A6. Surprisingly, on the 17th day, isolate A6, which displayed the largest clear zone diameter, was the one with the smallest clear zone diameter of 27.2 mm while the largest one, 32.2 mm, was displayed by isolate A3. Despite the unpredictable growth variation among three isolates of actinomycetes, isolate A3 which had the highest clear zone diameter on the 17th day was observed to consistently display the highest clear zone diameter from the 8th, 11th, and 14th days. After 13 days, most of the fungal colonies were bigger, covering the whole plates of which it was not possible to measure the diameter of clear zones. On the other hand, bacteria and actinomycetes had slow growth whereby after 17 days, most of the colonies were observed to degrade.

### 4.7 Comparative degradation of ground plastic bottles by fungi (5–13 days), bacteria, and actinomycetes (5–17 days) isolates

**FIGURE 4:**
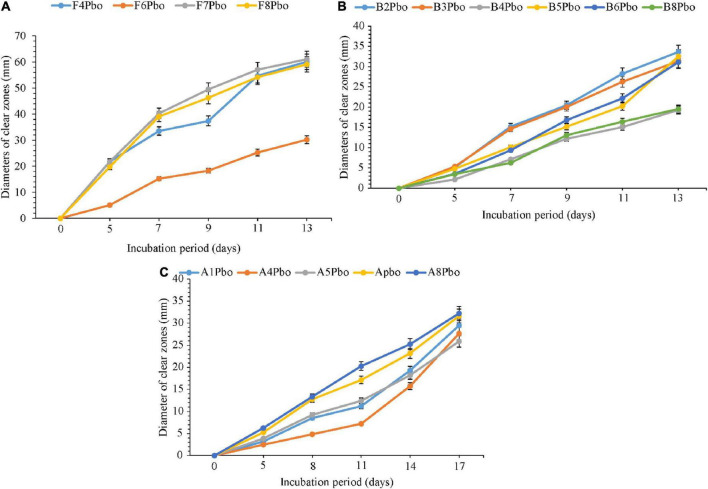
Comparative ability of the **(A)** fungi, **(B)** bacteria, and **(C)** actinomycetes in degrading ground plastic bottles. Error bars represent the mean ± SD of three independent biological replicates (the names of species coded with letters F represents fungi, B represents bacteria, and A represents actinomycetes, and the numbers represent their positions in a particular group as shown in Table 6).

The comparative degradation of plastic bags by fungi, bacteria, and actinomycetes is shown in Figure 4. There was a clear variation in the diameters of clear zones for fungi, bacteria, and actinomycetes in degrading ground plastic bottles on solidified Bushnell and Haas agar medium. There were differences in growth among species of fungi during the degradation of plastic bags from the 5th to the 13th day. The minimum diameter of 5.1 mm was observed in *Talaromyces islandicus* (F6) during the 5th day and the maximum diameter of 21.8 mm was observed in *Aspergillus terreus* (F4). The growth continued to vary from the 7th, 9th, and 11th days and, finally, on the 13th day, the minimum clear zone diameter was 30.2 mm, which was observed in the same isolate, which is *Talaromyces islandicus* (F6), and the maximum diameter was 61.1 mm, which is *Aspergillus terreus* (F4). *Aspergillus terreus* (F4) was the most efficient and *Talaromyces islandicus* (F6) was the least efficient in degrading the ground plastic bags. The minimum clear zone diameter of 2.1 mm recorded by bacteria on the 5th day was observed in *Sinomonas* sp. (B4) and the maximum, 5.3 mm, was observed in another isolate of *Sinomonas* sp. (B3). The variation in growth continued from the 8th, 11th, and 14th days; finally, on the 17th day, the minimum clear zone diameter of 19.3 mm was recorded in the same isolate *Sinomonas* sp. (B4) and the maximum, 33.6 mm, in *Bacillus cereus* (B2). This indicates that the bacterial *Bacillus cereus* (B2) was more efficient than other isolates in degrading ground plastic bottles. In the case of actinomycetes, the variation started from the 5th day as in fungi and bacteria whereby the minimum clear zone diameter of 2.5 mm was recorded in unsequenced isolate A4 while the maximum, 6.3 mm, was displayed by *Streptomyces rochei* (A8). Despite the variation in growth and formation of clear zones among the tested isolates of actinomycetes, *Streptomyces rochei* (A8) consistently displayed the highest clear zone diameter from the 5th to 17th days. The growth of fungi, bacteria, and actinomycetes followed the same trend as in plastic bags. There was an overgrowth of fungal colonies after 13 days and a degradation of colonies for bacteria and actinomycetes after 17 days.

### 4.8 Comparative efficiency of fungi, bacteria, and actinomycetes in degrading plastic bags and bottles

The comparative abilities of fungi, bacteria, and actinomycetes in degrading ground plastic bags and bottles in their final days of incubation were analyzed to assess whether the variation in the growth was either or not significant (Table 8). There was a significant (*p* < 0.001) variation in the abilities of the tested isolates in degrading both plastic bags and bottles. Unsequenced bacterial isolate, B1 from Kasanga 1 soil, was significantly (*p* < 0.001) effective in degrading the plastic bags as it displayed the largest clear zone diameter of 47.5 mm. On the other hand, *Bacillus cereus* (B2) isolated from SUA farm soil was significantly (*p* < 0.001) effective in degrading the ground plastic bottles by displaying the largest clear zone diameter of 33.6 mm. On the other hand, actinomycetes were significantly (*p* < 0.001) effective in degrading plastic bags and bottles. *Streptomyces weraensis* (A2) from Kasanga 3 soil displayed the largest clear zone diameter of 30.2 mm in degrading the plastic bags, and *Streptomyces rochei* (A8) from SUA farm soil displayed the largest clear zone diameter of 32.2 mm in degrading the plastic bottles. On the other hand, *Aspergillus* sp. (F7) from Kasanga 1 soil was significantly (*p* < 0.001) effective in degrading the plastic bags by displaying the largest clear zone diameter of 61.1 mm. In contrast, *Phoma* sp. (F2) isolated from Kasanga 1 soil was significantly (*p* < 0.001) more effective in degrading the plastic bottles than the rest of the fungal species.

**TABLE 8:** Comparative efficiency of different bacteria, actinomycetes, and fungi in degrading polyethylene plastics bag and bottle on the 13th (fungi) and 17th days (bacteria and actinomycetes).

Isolate	Location	Degradation ability, clear zone diameter (mm)	Type of plastic degraded
**Bacteria on 17th day**			
B1	Kasanga 1	47.5 ± 0.01^b^	Plastic bag
B7	Kasanga 3	21.7 ± 0.02^a^	Plastic bag
*Bacillus cereus* (B2)	SUA farm	33.6 ± 0.02^d^	Plastic bottle
*Sinomonas* sp. (B3)	SUA farm	31.4 ± 0.02^b^	Plastic bottle
*Sinomonas* sp. (B4)	SUA farm	19.3 ± 0.03^a^	Plastic bottle
*Cellulosimicrobium* sp. (B5)	Kasanga 3	32.5 ± 0.02^c^	Plastic bottle
*Cellulosimicrobium* sp. (B6)	Kasanga 3	31.1 ± 0.02^b^	Plastic bottle
B8	Kasanga 1	19.6 ± 0.01^a^	Plastic bottle
**Actinomycetes on 17th day**			
*Streptomyces weraensis* (A2)	Kasanga 3	30.2 ± 0.03^b^	Plastic bag
A3	Kasanga 1	32.2 ± 0.02^c^	Plastic bag
A6	Kasanga 2	27.2 ± 0.02^a^	Plastic bag
A1	SUA farm	29.5 ± 0.01^c^	Plastic bottle
A4	SUA farm	27.6 ± 0.02^b^	Plastic bottle
A5	Kasanga 2	25.9 ± 0.01^a^	Plastic bottle
A7	Kasanga 3	31.6 ± 0.01^d^	Plastic bottle
*Streptomyces rochei* (A8)	SUA farm	32.2 ± 0.03^e^	Plastic bottle
**Fungi on 13th day**			
*Aspergillus terreus* (F4)	Kasanga 2	60.1 ± 0.02^c^	Plastic bag
*Talaromyces islandicus* (F6)	SUA farm	30.2 ± 0.03^a^	Plastic bag
*Aspergillus* sp. (F7)	Kasanga 1	61.1 ± 0.01^d^	Plastic bag
*Aspergillus terreus* (F8)	SUA farm	59.1 ± 0.02^b^	Plastic bag
*Eupenicillium rubidurum* (F1)	Kasanga 3	46.1 ± 0.02^b^	Plastic bottle
*Phoma* sp. (F2)	Kasanga 1	66.3 ± 0.03^d^	Plastic bottle
*Neosartorya fischeri* (F3)	Kasanga 3	48.3 ± 0.02^c^	Plastic bottle
*Aspergillus terreus* (F5)	Kasanga 2	33.3 ± 0.04^a^	Plastic bottle

Different letters represent the significantly at *P* = 0.05.

## 5 Discussion

The microbial populations in all soils varied from as low as 1.60 × 10^4^ CFU/g soil (fungi, Kasanga 3 soil) (4.20 log CFU/g soil) to 1.57 × 10^5^ CFU/g soil, which is the population of actinomycetes from SUA farm soil (5.20 log CFU/g soil). These results seem to indicate a relatively low capacity of the soils to sustain higher microbial populations above 10^5^ CFU/g of soil. Similar observations were made by Akande and Adekayode (2019) while comparing the levels of microbial populations isolated from different types of soils.

Under the microscopic observations, some actinomycete isolates developed a black substrate mycelium with sporulated aerial mycelium. These observations are similar to the phenomenon explained by Ng et al. (2013) that *Streptomyces* can form a non-fragmenting substrate mycelium that may bear spores, and in most genera, a well-developed aerial mycelium with spore chains that can be long or very short. The microscopic features observed for the fungi were conidiophores, conidia, and phialides, which are the common characteristics of *Aspergillus* and *Penicillium* (Golding et al., 2016).

The identified bacterial species included *B. cereus, Sinomonas* sp., and *Cellulosimicrobium* sp. while actinomycetes included *S. werraensis* and *S. rochei*. The fungal isolates were identified as *E. rubidurum, Phoma* sp., *N. fischeri, A. terreus*, and *T. islandicus*. *Aspergillus terreus* appeared in two different soils of the Kasanga and SUA farm. Differences were observed among these microorganisms in their comparative abilities to degrade the ground PE plastic bags. This observation is in line with that of Gajendiran et al. (2016) who identified five bacterial species, including *Streptococcus* and *Staphylococcus*, and eight fungal species of *Aspergillus*. Different genera/species of bacteria and *Streptomyces* degrade the plastics such as PE (Gupta and Devi, 2020). In the study by Deepika and Madhuri (2015), for instance, significant differences in weight loss of LDPE as compared to initial weight were attributed to their degradation by *Pseudomonas* sp., *A. niger*, and *A. flavus*. On screening of the ability of different microorganisms in degrading polyethylene, fungi were more efficient than bacteria and actinomycetes. Shah et al. (2016) also observed the ability of *B. subtilis* to degrade polyurethane. Various research studies have also reported on the abilities of different genera/species of fungi on degrading different types of plastics. Raaman et al. (2012) reported the biodegradation of plastics by *Aspergillus* spp., including *A. terreus* isolated from polythene-polluted sites around Chennai in India. Other studies have observed *Eupenicillium* sp., *Talaromyces* sp., and *Penicillium simplicissimum* to have the ability to degrade PE (Sowmya et al., 2015). In general, the results presented in Table 6 on the involvement of different genera/species further confirm the diversity of bacteria, fungi, and actinomycetes genera and/or species that can degrade plastics. Other studies have similarly shown that different genera/species of bacteria, including *Pseudomonas* sp. (Nanda et al., 2010), *Streptococcus* spp., *Staphylococcus* spp., *Micrococcus* spp. and *Moraxella* spp., *B. subtilis*, *B. amylolyticus*, and *Arthrobacter defluvii* (Prabhat et al., 2013), actinomycetes, *Streptomyces* sp. (Deepika and Madhuri, 2015), and fungi, including *A. niger*, *A. japonicus*, *A. terreus, A. flavus*, and *Mucor* sp. (Ibrahim et al., 2011; Raaman et al., 2012), exhibited the ability to degrade LDPE.

Differences in the abilities of various microorganisms to degrade plastics might be due to the differences in environments from where they are isolated. However, the main mechanisms for microbial degradation of PE plastics are oxidation of the PE surface and formation of carbonyl groups, which cause deterioration and fragmentation of the material (Ali et al., 2022). Nanda et al. (2010) investigated the same phenomenon by comparing three *Pseudomonas* sp. from three different isolation sources, namely, sewage sludge dump, household garbage dump, and textile effluents drainage site. They observed that *Pseudomonas* sp. from sewage sludge dump degraded polyhydroxyalkanoate (PHA), a natural plastic, more efficiently by 46.2%, as compared to its ability to degrade by 29.1% of a synthetic PE. In contrast, *Pseudomonas* sp. from the household garbage dump gave the lowest biodegradability of 31.4% and 16.3% for the natural plastic and synthetic PE, respectively. However, *Pseudomonas* sp. isolated from the textile effluent drainage site gave an intermediate biodegradability of 39.7 and 19.6% for the natural plastic and synthetic PE, respectively. Thus, the differences in plastic biodegradation between and within species of a microorganism, as presently observed using the diameters of the clear zones, can always be expected.

The growth rate of the microorganisms was slow (2–3 days), although the nutrients were amply available. This indicates that the organisms were not yet well-adapted to the available carbon source, plastics. The growth of bacteria and actinomycetes from the 5th to the 13th days and up to the 17th day for fungi increased, implying that microorganisms had adapted to the available foreign carbon source. The degradation rate of *Pseudomonas* sp., increased until the 21st day after which it took a sudden deep (Nanda et al., 2010), implying that *Pseudomonas* sp. had metabolized the available basal media nutrients before utilizing the carbon sources from the PE. The degradation of the colonies for bacteria and actinomycetes is an implication that the microbe cannot reach the available carbon beyond the clear zone. The microorganisms differ in their speed of degradation of plastics, and this might be due to the differential capacity of the enzymes produced to catalyze the degradation of the plastics (Shah et al., 2008). This demonstrates that biodegradation is dependent on polymer characteristics, organism type, and nature of pre-treatment. For the case of this study, the maximum time for growth was 21 days observed in *Pseudomonas* sp.

It should be noted that not all species occurring in nature exhibit the ability to degrade plastics. This is because they might be different/distinct strains. For example, two isolates of *A. terreus* were isolated from Kasanga 2 soil: *A. terreus*-F4 degraded ground plastic bottle and *A. terreus*-F5 degraded a ground plastic bag. Another isolate of *A. terreus*-F8 was isolated from the SUA farm, and it degraded the ground plastic bottle. These may not be the same strain. It is also possible that a given organism, for example, *A. terreus*, will have other strains that have a greater ability to degrade a given type of plastic while others do not. Ibrahim et al. (2011) observed strains of *A. terreus* that degraded PE by 58.0% as tested using colony diameter on Petri dishes. This is an indication that while the organism used in these two different studies cited was *A. terreus*, they could be two distinct strains as depicted by their huge difference in the extent of their degradation of PE. Therefore, it should not be assumed that any isolates of the same genus/species automatically have the equal capability of degrading a given plastic.

## 6 Conclusion

The higher demand for single-use plastics and their disposal in the environment is a serious environmental polluting component. The available management strategies have been used for many years, but still, the yield of plastic pollutants is increasing in the environment. It is high time to explore the new sustainable and environmentally safe technique of plastic degradation by beneficial soil microorganisms in removing the plastic pollutants from the environment. The soils used in this study contained microorganisms that were capable of degrading ground PE plastic bags and bottles as indicated by large clear zones of up to 66.3 mm by *Phoma* sp. (F2) in degrading the plastic bottles and 61.1 mm by *Aspergillus* sp. (F7) in plastic bags within 13 days. Moreover, this study revealed that the microbial degradation of plastics is widespread, among bacteria, fungi, and actinomycetes. Different genera/species including *Bacillus cereus* (bacteria), *Streptomyces werraensis* (actinomycete), *Aspergillus*, and *Phoma* sp. (fungi) that degrade PE plastics were identified in this study. Some could not be identified currently, calling for further study on them. The identified isolates especially the highly efficient ones hold the potential to be exploited industrially (in fermenters) or environmentally (in landfills) to degrade the waste plastics. Therefore, bioprospecting of agricultural soils, water bodies, and landfills containing plastic wastes could lead to the identification of more efficient microbial species with the ability to degrade plastics.

## Data availability statement

The datasets presented in this study can be found in online repositories. The names of the repository/repositories and accession number(s) can be found in this article.

## Author contributions

MN: original draft preparation. HT, GM, and ES: review and editing. All authors: conceptualization, methodology, experiments, read, and agreed to the published version of the manuscript.
